# Graphene oxide‐ellagic acid nanocomposite as effective anticancer and antimicrobial agent

**DOI:** 10.1049/nbt2.12009

**Published:** 2021-02-02

**Authors:** Samer Hasan Hussein‐Al‐Ali, Suha Mujahed Abudoleh, Mohd Zobir Hussein, Saifullah Bullo, Arul Palanisamy

**Affiliations:** ^1^ Department of Chemistry Faculty of Science Isra University Amman Jordan; ^2^ Faculty of Pharmacy Isra University Amman Jordan; ^3^ Materials Synthesis and Characterization Laboratory Institute of Advanced Technology (ITMA) University Putra Malaysia Selangor Malaysia; ^4^ Laboratory of Vaccines and Immunotherapeutics Institute of Bioscience University Putra Malaysia Selangor Malaysia

## Abstract

In this study, ellagic acid (ELA), a skin anticancer drug, is capped on the surface(s) of functionalised graphene oxide (GO) nano‐sheets through electrostatic and π–π staking interactions. The prepared ELA‐GO nanocomposite have been thoroughly characterised by using eight techniques: Fourier‐transform infrared spectroscopy (FTIR), zeta potential, X‐ray diffraction (XRD), thermogravimetric analysis (TGA), Raman spectroscopy, atomic force microscopy (AFM) topographic imaging, transmission electron microscopy (TEM), and surface morphology via scanning electron microscopy (SEM). Furthermore, ELA drug loading and release behaviours from ELA‐GO nanocomposite were studied. The ELA‐GO nanocomposite has a uniform size distribution averaging 88 nm and high drug loading capacity of 30 wt.%. The in vitro drug release behaviour of ELA from the nanocomposite was investigated by UV–Vis spectrometry at a wavelength of *λ*_max_ 257 nm. The data confirmed prolonged ELA release over 5000 min at physiological pH (7.4). Finally, the *IC*_50_ of this ELA‐GO nanocomposite was found to be 6.16 µg/ml against B16 cell line; ELA and GO did not show any cytotoxic effects up to 50 µg/ml on the same cell lines.

## 1 INTRODUCTION

Ellagic acid (ELA) is an organic hetero‐tetra‐cyclic compound with planar phenolic lactone properties. It is found in many fruits and vegetables, such as pomegranates, cranberries, strawberries and raspberries. It has a role as a natural phenol antioxidant, which is a group of anticancer drugs [1,2]. ELA is usually inserted into drug delivery vehicles including zinc layered hydroxides [3], silver nanoparticles [4], metalla‐cages [5], polylactide glycolide (PLGA) nanoparticles [6], and chitosan nanoparticles [7].

Development of research on nanomaterials for medical applications has been carried out in the last three decades; most researches concentrated on their safety and toxicity on human cell and tissue function [8, 9, 10, 11]. However, the biomedical applications of GO, such as anticancer drug delivery, developed quickly over the past 15 years. Graphene oxide (GO) has been extensively researched as a promising bio‐carbon‐material for different medical applications due to their exclusive properties: two‐dimensional planar structure with *sp* ^2^ hybridised carbon, large surface area with different functional groups (hydroxyl, carboxylic groups and epoxide) [12], chemical stability and excellent biocompatibility. These properties lead to exclusive applications for drug delivery systems [13,14]. Recently, different attempts have been made to incorporate different compounds for the preparation of soluble graphene for drug delivery such as the incorporation of 1‐aminooctadecane [15], isocyanatobenzene [16], 5‐(4‐aminophenyl)‐10,15,20‐(triphenyl)porphyrin [17], doxorubicin hydrochloride [18], and docetaxel [19].

In this present study, the anticancer drug ELA was chosen as a model using modified GO. These new nanocomposite were characterised and applied on B16 cancer cell lines and different microorganisms to check their drug activity.

## 2 MATERIALS

Graphite (Gr), ELA (97% purity), phosphate‐buffered saline (PBS) and modified Dulbecco’s medium (DMEM) were purchased from Sigma‐Aldrich (St Louis, MO, USA); potassium permanganate (KMnO_4_, 99%), sulphuric acid (H_2_SO_4_, 95%–97%), diethyl ether (C_2_H_5_)_2_O, hydrochloric acid (HCl, 37%), hydrogen peroxide (H_2_O_2_, 35%), and o‐phosphoric acid (H_3_PO_4_, 85%) were obtained from Friendemann.Schmidt. The Cell Counting Kit‐8 (CCK‐8) used was manufactured by Dojindo Lab (Japan), and the 96‐well microplates were purchased from Corning Technologies (Corning, NY, USA).

The bacterial strains used were obtained from the Faculty of Medicine at the University of Jordan (Jordan). The bacterial strains studied are *Proteus mirabilis* (ATCC12453), methicillin‐resistant *Staphylococcus aureus* (MRSA) (ATCC43300), *Pseudomonas aeruginosa* (ATCC27853), *Klebsiella pneumoniae* (ATCC13883), *Escherichia coli* (ATCC8739) and *Staphylococcus aureus* (ATCC33862).

## 3 METHODS

### 3.1 Convert graphite to graphene oxide

Graphite (Gr) was converted to GO using a modified Hummers method [20]. The stepwise preparation is as follows:

1. The Gr flakes (3 mg) and KMnO_4_ (38 g) were mixed in 360 ml of H_2_SO_4_ (98%) and 40 ml of H_3_PO_4_ in a 1000 ml‐volumetric flask and kept in an ice bath.
2. The mixture was stirred continuously for 12 h with heating at 50 C and then cooled to room temperature.
3. The content of the reaction was poured into 400 ml ice and 3 ml H_2_O_2_ (30%), and centrifuged at 4000 rpm for 3 min. The washing was carried out using water, 200 ml HCl (37%) and 200 ml ethanol.
4. The coagulation process occurred using 200 ml diethyl ether.
5. Finally, the GO was filtered by Ommipore membrane.

### 3.2 Preparation of the ellagic acid‐graphene oxide nanocomposite

The ELA‐GO nanocomposite were prepared as follows: 0.3410 g of ELA was dissolved in 50 ml of dimethyl sulfoxide (DMSO), stirred and heated up to 40°C. After that, 0.2 g of GO were mixed with the ELA solution, and the pH was adjusted to 4.7 using NaOH. The reaction contents were stirred for 18 h. The final product was filtered and washed with deionised water five times; the nanocomposite was dried in an oven for 12 h.

### 3.3 Loading of ellagic acid into and release from ellagic acid‐graphene oxide nanocomposite

The amount of ELA loaded into the ELA‐GO nanocomposite was determined by separating the ELA solution from GO. Different terms are used during the calculation; *T*_t_ is the initial mass of drug used in the experiment, *T*_f_ is the mass of drug in supernatant. Therefore *T*_t_–*T*_f_ represents the mass of drug binding in the formulation.

The procedure for determination of *T*_f_ was carried out by centrifuge the nanocomposite at 4000 rpm for 10 min and the absorbance was measured. The mass of unbinding ELA in the supernatant was measured through the calibration curve.

The mass of the nanocomposite was determined after the centrifugation, washing and drying the formulation. All the previous steps were measured by a UV spectrophotometer at a wavelength of 257 nm and used in the following Equation (1):

(1) $$\text{Loading}\;\text{Effciency}\%=\frac{T_{\text{t}}-T_{\text{f}}}{\text{mass}\;\text{of}\;\text{nanocomposite}}\times 100$$

The ELA release from ELA‐GO nanocomposite was determined by using a PBS solution similar to physiological body pH (pH 7.4). About 100 mg of the ELA‐GO nanocomposite was added to 500 ml of PBS. About 2 ml from release medium was removed at different times and exchanged with 2 ml of the buffer. The cumulative amount of ELA free in the solution was measured at *λ*_max_ = 257 nm using a Shimadzu UV‐1601 spectrophotometer instrument. To compare the release behaviour of ELA from ELA‐GO with that from the physical form, the 30 mg of ELA was mixed with 70 mg of GO and determined by a phosphate‐buffered solution.

### 3.4 Cell culturing and cytotoxicity assays

The CCK‐8 assay was used to measure the cell viability in a microplate reader. The B16‐F10 and 3T3 cell lines were harvested in 96‐multi‐well plates at a cell density of 1.5 × 10^5^ cells/ml in 100 μl of Roswell Park Memorial Institute (RPMI) 1640 medium which contained DMEM supplemented with 10% heat‐inactivated fetal bovine serum (FBS). The old media was removed, and then, 100 μl of the new DMEM medium containing ELA, GO, and ELA‐GO nanocomposite was used to treat the B16‐F10 and 3T3 cells. The control wells contained only media and cells. A 10 μl sample of CCK‐8 assay was added to the wells under conditions of 37°C in a 5% carbon dioxide humidified incubator. The absorbance of each well was measured using a microplate reader at *λ*_max_ = 450 nm. The cell viability was calculated according to the literature [21].

### 3.5 Test material preparations for microorganisms

The ELA was dissolved in DMSO to final concentrations of 5, 1 and 0.5 mg/ml. The suspension of GO was prepared in 0.9% PBS into a final concentration of 5 mg/ml. The ELA‐GO nanocomposite was prepared as a suspension in 0.9% normal saline into final concentrations of 5, 1, and 0.5 mg/ml. The DMSO alone was used as a negative control.

### 3.6 Antibacterial assay

The antibacterial activity of ELA‐GO nanocomposite was evaluated by the agar well diffusion method using nutrient agar medium for the assay. The microorganism under study was activated by inoculating a loopful of the strain in 10 ml of the nutrient broth and incubated at 37 C in an orbital shaker. The density of each of the bacterial strains was adjusted to 0.5 McFarland. 100 μl of each McFarland was spread over the surface of nutrient agar. After that, a well was made in the seeded plates with the help of an 8 mm cup‐borer. 100 μl of the test materials was introduced into the wells, followed by incubation at 37 C for 24 h. The antibacterial activity was tested in triplicates by measuring the diameter of the zone of inhibition.

## 4 INSTRUMENTATION

All the prepared samples were measured using different instruments described as follows:

1. Powder X‐ray diffraction (PXRD) in the range of 2°–40°  by 6000 diffractometer model (Shimadzu, Tokyo, Japan) with CuK (alpha) X‐ray sources (λ 1.54 Å) at 30 kV and 30 mA.
2. Fourier‐transform infrared spectroscopy (FTIR) data spectra of the ELA, GO and ELA‐GO nanocomposites were measured in the range of 400–4000 cm^−1^ wave number on a Thermo Nicolet Nexus, Smart Orbit spectrometer.
3. TGA was conducted using a Metter‐Toledo 851e instrument (Switzerland) at a heating rate of 10°C/min from 30°C to 900°C.
4. Transmission electron microscope imaging of the GO and ELA‐GO nanocomposites were studied using FEI model (USA).
5. Raman imaging spectra were measured using a UHTS 300 model (WITec, Germany) with excitation at 532 nm.
6. The zeta potential (*ζ*) was determined at room temperature by dynamic light scattering using a Malvern Zetasizer Nano ZS instrument (United Kingdom).
7. The ultraviolet–visible (UV–Vis) spectra were measured to determine the controlled release study, using a UV–Vis spectrophotometer (Shimadzu UV‐1601).

## 5 RESULTS OF THE DATA AND DISCUSSION

### 5.1 Powder X‐ray diffraction

**FIGURE 1:**
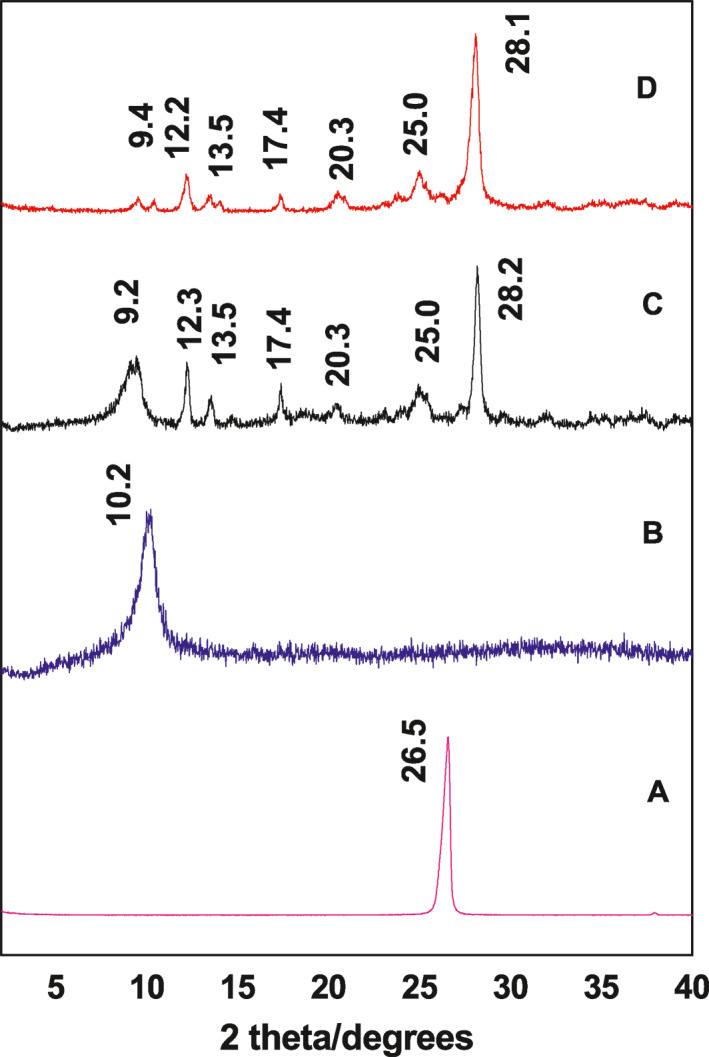
Powder X‐ray diffraction (PXRD) patterns of the Gr (a), graphene oxide (GO) (b), ellagic acid (ELA)‐graphene oxide (GO) nanocomposite (c) and ELA drug (d)

Figure 1a–d shows the PXRD patterns of Gr, GO, ELA‐GO nanocomposite and ELA, respectively. The PXRD diffraction peak of pure graphite (Figure 1a) is found around 26^o^ due to the highly layered structure with a 0.34 nm interlayer distance along the (002) orientation. Figure 1b shows the diffraction angle 2*θ* = 10^o^, which is mostly due to the conversion of Gr to GO after the chemical oxidation and exfoliation. At the same time, the 0.34 nm interlayer distance of Gr changed to 0.82 nm, indicating the formation of GO.

Figure 1d shows the PXRD diffraction patterns with a sharp peak of ELA at 9.4 , 12.2 , 13.5 , 17.4 , 20.3 , 25.0 , and 28.1  [2]. The ELA‐GO nanocomposite (Figure 1c) shows an amorphous characteristic, with the presence of crystalline peaks of ELA. This result may be due to the presence of free drug in the prepared sample and loaded ELA on the GO carrier. Moreover, the result shows that there is no large change between the diffraction patterns of GO and ELA‐GO nanocomposites (the diffraction peak at 2*θ* = 10^o^), proving that the loading of ELA did not disturb the GO nanocarrier.

### 5.2 Infrared spectroscopy

**FIGURE 2:**
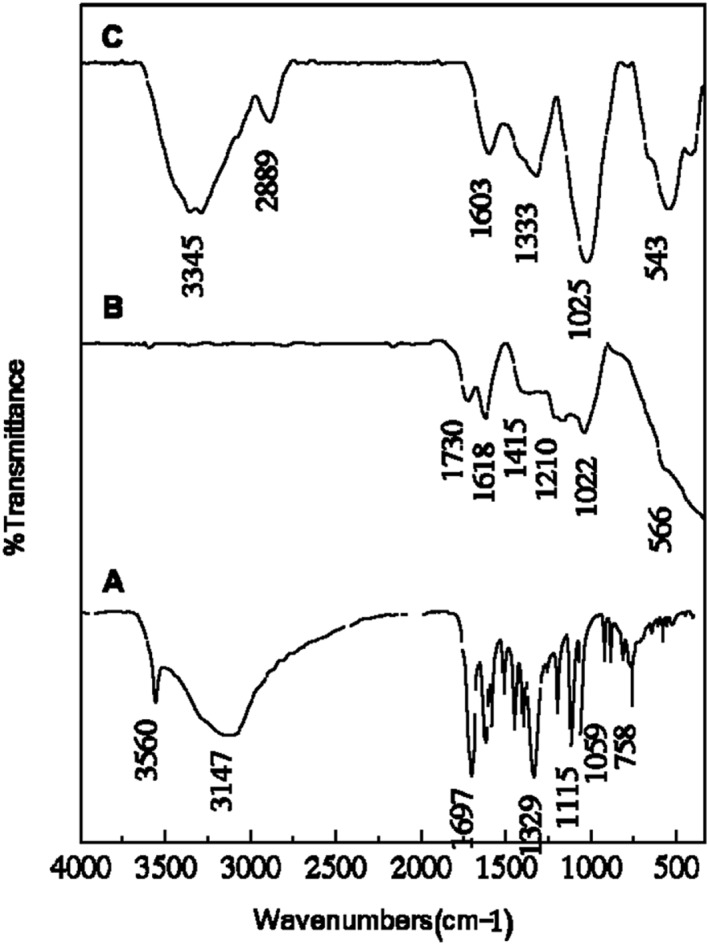
Fourier‐transform infrared spectroscopy (FTIR) analysis of the ellagic acid (ELA) (a), graphene oxide (GO) (b), and ELA‐GO nanocomposite (c)

Figure 2 shows the FTIR spectra analysis of ELA, GO, and ELA‐GO. For ELA (Figure 2a), the infrared (IR) characteristic peak of O–H was in the range of 2800–3700 cm^−1^ [7], and the IR characteristic peak of C=O stretching was at 1697 cm^−1^ [7,22,23]. The characteristic peaks shown between 1618 and 1510 cm^−1^ are due to a C=C aromatic ring while characteristic peaks at 1196 and 1059 cm^−1^ are due to –COO‐C ester linkage [7,22,23]. The characteristic peak at 751 cm^−1^ is due to C‐H aromatic bending.

The FTIR spectrum of GO nanosheets is depicted in Figure 2b. The spectrum shows IR characteristic peak of C=O stretching of carboxylic and/or carbonyl moiety functional groups at 1730 and 1618 cm^−1^ [24] and an IR characteristic peak of C–O stretching vibrations at about 1210 and 1022 cm^−1^. This carbonyl group (C=O) in GO would assist the connection of nanoparticles such as gold nanoparticles or protein and DNA through an electrostatic interaction or covalent bond [12,13].The characteristic peak of ELA at 1333 and 543 cm^−1^ was clearly observed in Figure 2c, with a slight shift. This suggested that ELA was successfully conjugated on the GO nanosheets.

### 5.3 The interaction between ellagic acid and graphene oxide in the ellagic acid‐graphene oxide nanocomposite

**FIGURE 3:**
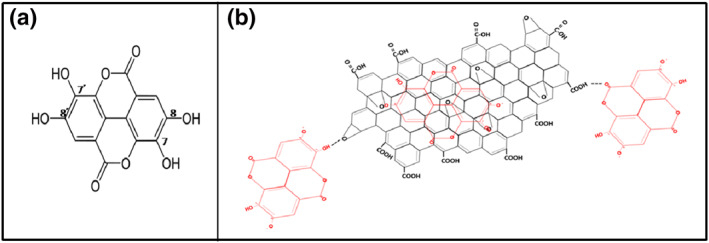
The interaction between ellagic acid (ELA) and graphene oxide (GO) in the ELA‐GO nanocomposite

The ELA is a planar phenolic lactone with two deprotonated hydroxyl groups at position eight and/or 8̀ at pH larger than 5.6 (Figure 3a) [22]. Therefore, the ELA drug was loaded onto the surface of GO by hydrophobic interactions and π–π aromatic rings stacking [25]. Two‐sandwich structures were opted for in this study, including GO‐ELA‐GO and ELA‐GO‐ELA (Figure 3b) [26].

### 5.4 Determination of the zeta potential (ζ)

**FIGURE 4:**
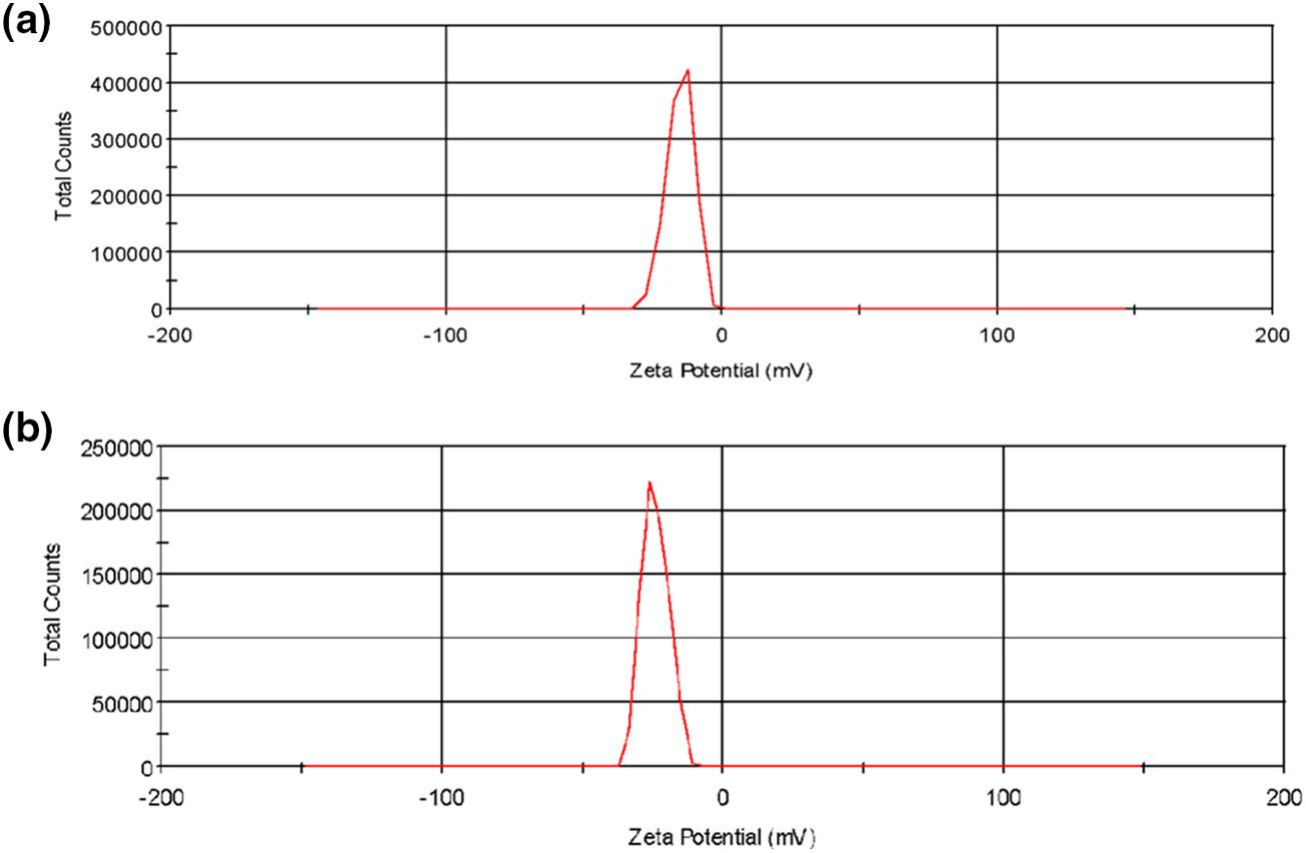
Zeta potential measurements of graphene oxide GO (a) and ellagic acid (ELA)‐GO nanocomposite (b)

Figure 4a,b shows the zeta potential (*ζ*) measurements of the GO and ELA‐GO nanocomposite, respectively. From the literature, GO nanosheets are stable in the pH range of 3–12 because their zeta potential is lower than ‐30 mV, which prevents the GO nanosheets from aggregating through electrostatic repulsion [27]. A GO nanosheets exhibits a zeta potential of about ‐15 mV, which is due to occurrence of carboxyl groups (COOH) [18]. After loading the ELA drug, the zeta potential (*ζ*) of the nanocomposite became even more negative with a value of ‐24 mV, which is appropriate to the larger negatively charged ELA on the surface of GO nanosheets [28].

### 5.5 Determination of the size distribution properties

**FIGURE 5:**
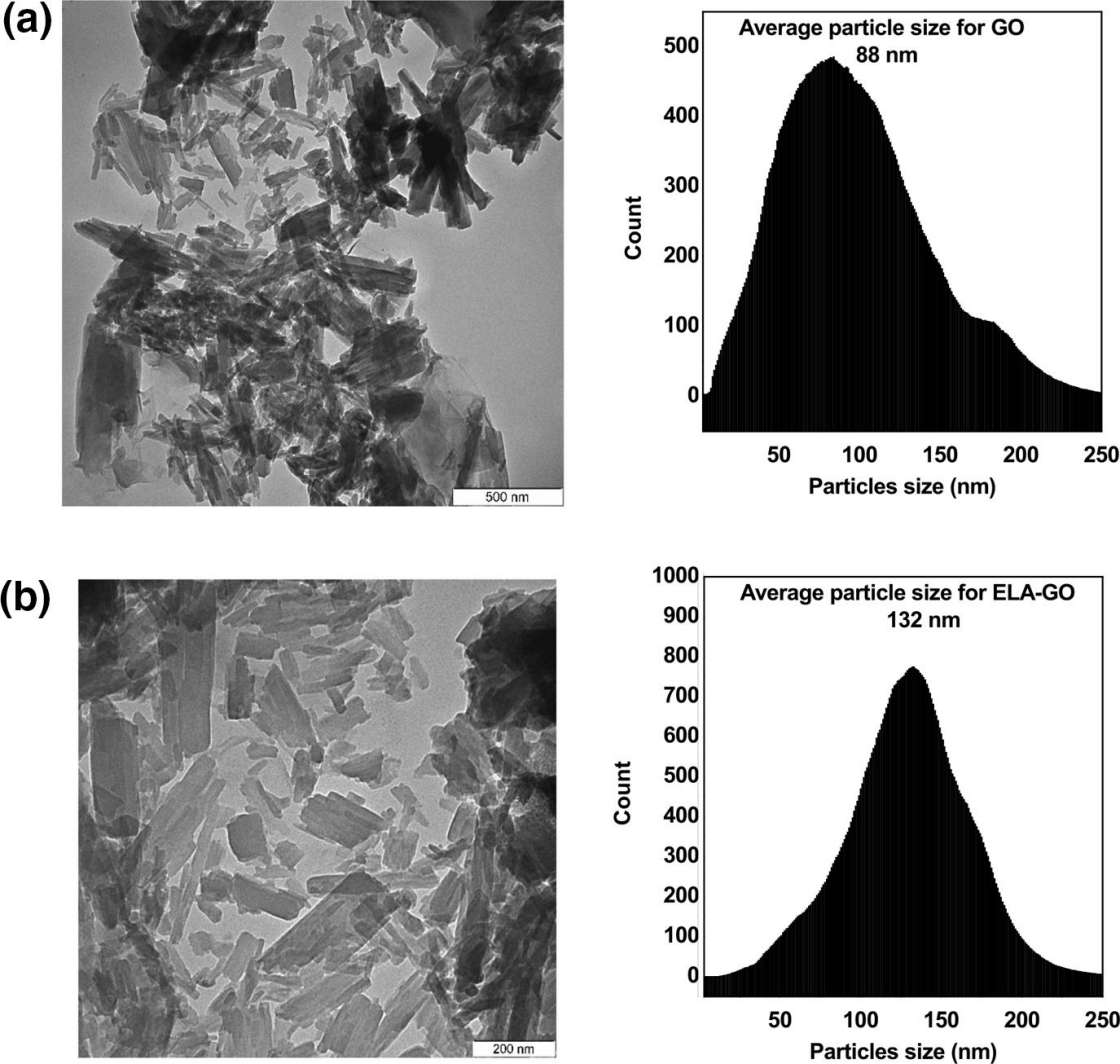
Transmission electron microscope images of graphene oxide (GO) (a), and ellagic acid (ELA)‐GO nanocomposite (b)

Transmission electron microscope imaging was used to characterise the morphology of the GO and ELA‐GO nanocomposite. Figure 5a shows a GO nanocarrier multilayered agglomerate [21,29]. The transmission electron microscope images of ELA‐GO nanocomposite in Figure 5b also show agglomerate multilayer sheets. The samples of GO and ELA‐GO show an average size of 88 and 132 nm, respectively. These average sizes were calculated using Image J software [30].

### 5.6 Scanning electron microscopy analysis

**FIGURE 6:**
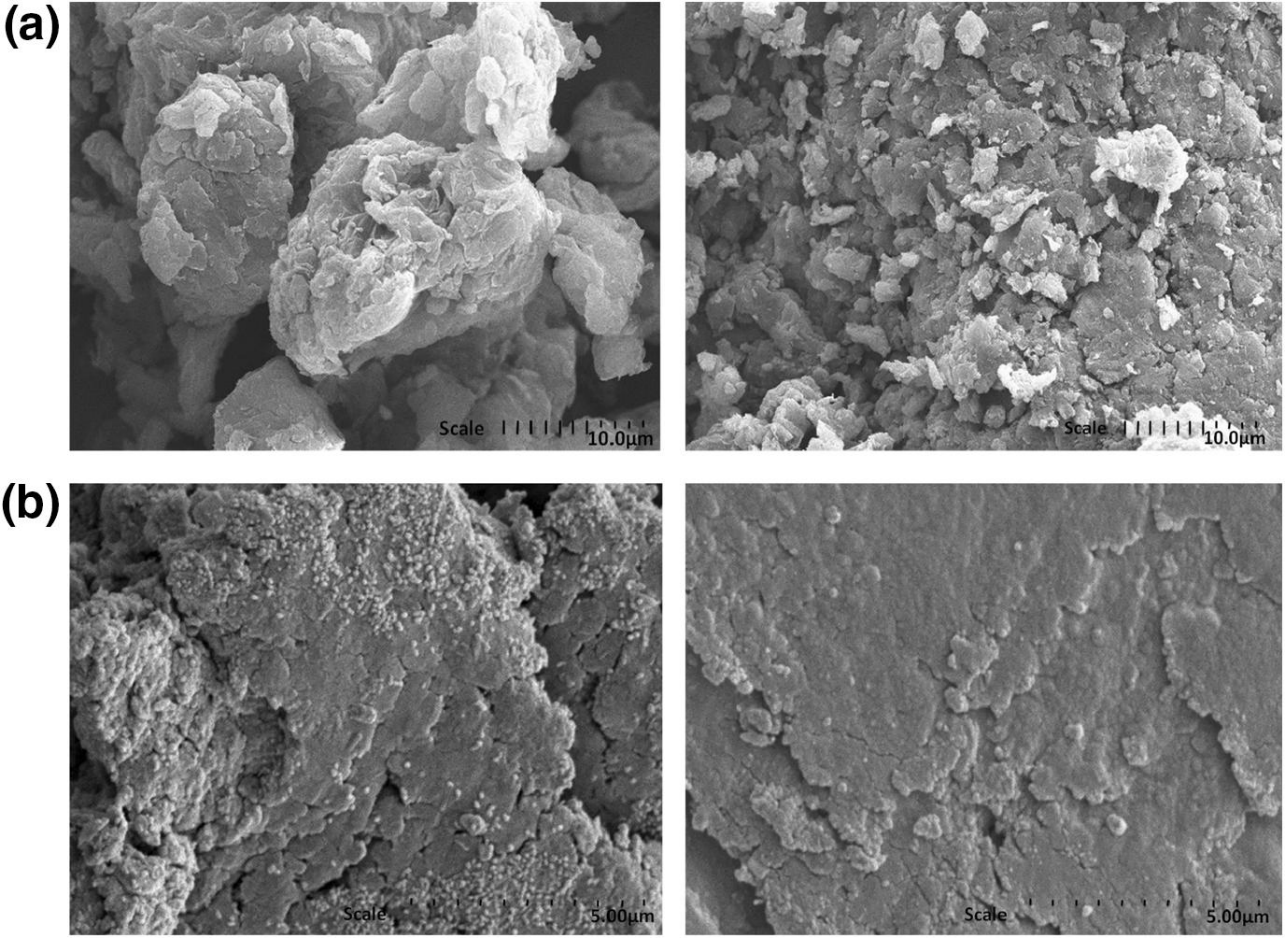
SEM images of graphene oxide (GO) (a), and ellagic acid (ELA)‐graphene oxide (GO) nanocomposite (b)

Figure 6a,b shows the SEM images of the GO and ELA‐GO nanocomposite at 10,000× magnification, respectively. From Figure 6a, it is clear that the sheets are stacked together with exfoliation [31], whereas the morphology of the ELA‐GO nanocomposite was loose but porous in Figure 6b [32].

### 5.7 Thermogravimetric analysis

**FIGURE 7:**
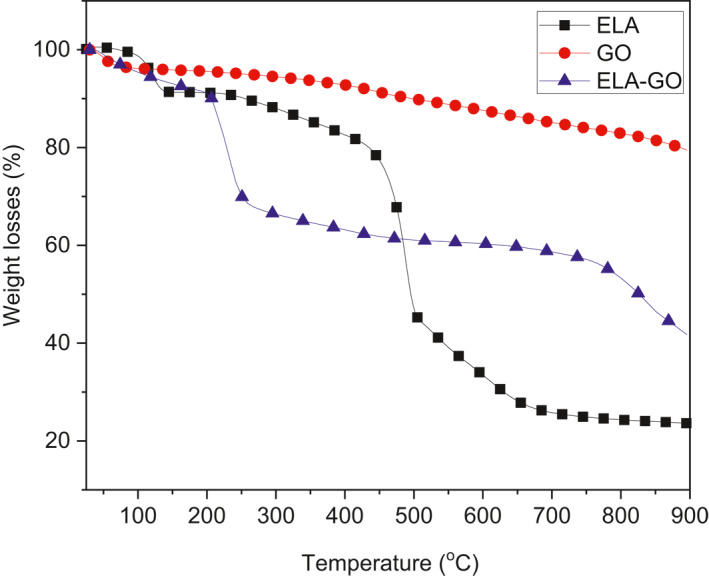
hermogravimetric analysis (TGA) curves are shown for ellagic acid (ELA), graphene oxide (GO), and ELA‐GO nanocomposite

The TGA thermograms of ELA, GO and ELA‐GO nanocomposite are shown in Figure 7. The results from TGA showed that there was a significant mass loss for GO powders. The mass loss percent below 100°C was related to the evaporation of H_2_O, while loss that occurred between 120°C and 260°C was related to decomposition of the oxygen functional groups, forming CO and CO_2_ gases [33]. TGA obtained for ELA showed three mass losses; the first mass loss had a value of 8.2% at maximum 112°C and was due to the removal of bonded H_2_O with hydrogen bond. The second and third mass losses of ELA occurred at 463 C (39%) and 596 C (17.5%). Because ELA‐GO nanocomposite are mainly composed of ELA, their TGA curves are like those of the pristine ELA and have the most significant mass loss between 200°C and 900°C, corresponding to decomposition of ELA. The mass losses for the GO was less than the ELA‐GO, thus confirming that the ELA was loaded onto the surface of GO.

### 5.8 Raman spectroscopy analysis

**FIGURE 8:**
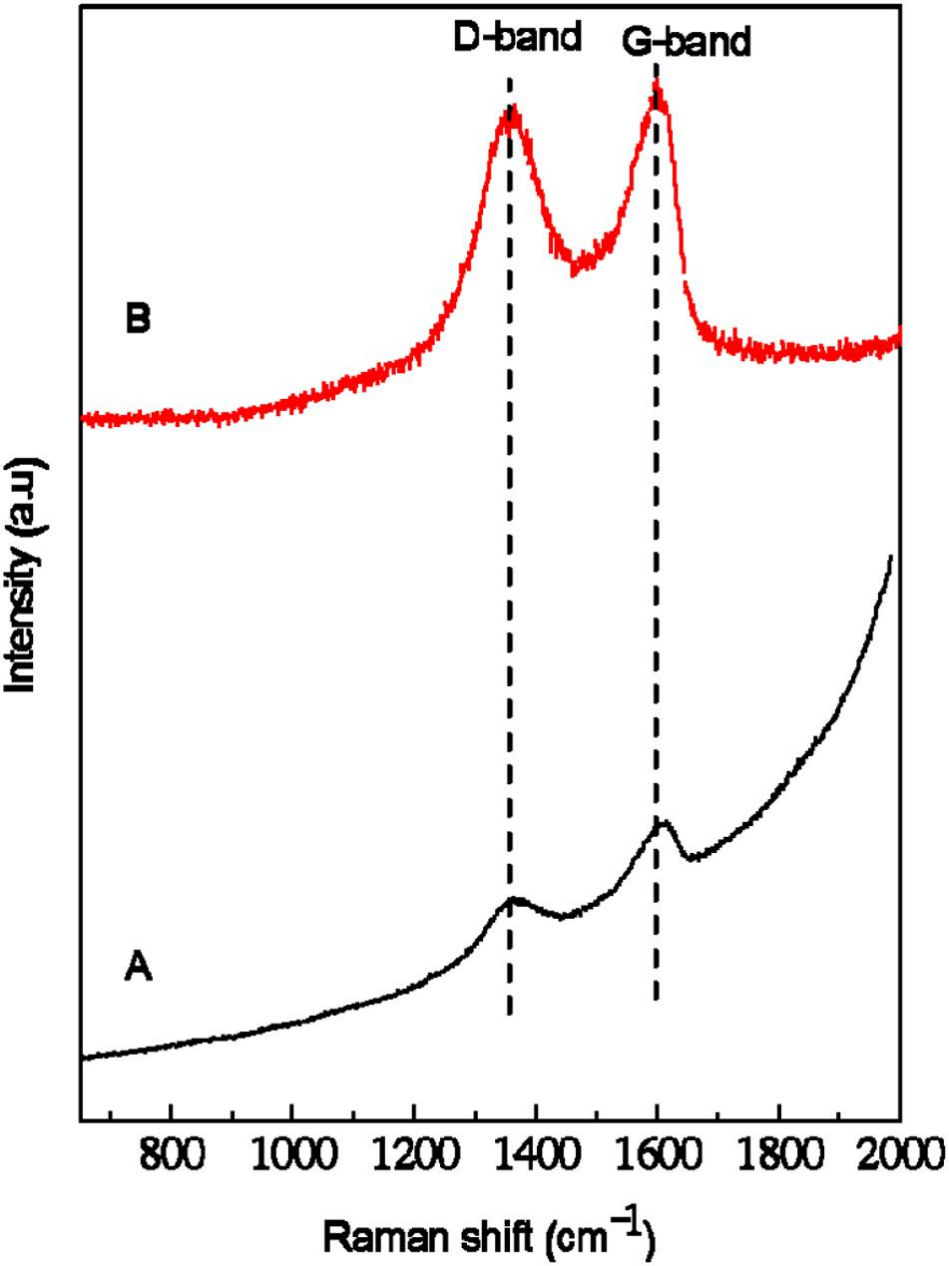
Raman spectra of graphene oxide (GO) (a), and ellagic acid (ELA)‐GO nanocomposite (b)

Raman spectroscopy is a beneficial tool used to describe the structure of GO nanosheets. The Raman spectra of GO and ELA‐GO nanocomposite in the spectral region 650–2000 cm^−1^ are presented in Figure 8. In Figure 8a, two fundamental vibrations are shown for G‐band at 1607 cm^−1^ and D‐band at 1364 cm^−1^.The G and D bands were due to the first‐order scattering of the E_2g_ mode and *k*‐point photons of A1g symmetry, respectively [34]. However, for the ELA‐GO nanocomposite, the characteristic G and D bands appear at 1600 and 1363 cm^−1^, respectively. The D/G intensity ratio of the ELA‐GO (0.98) increased compared to that of GO (0.76), which indicates that the *sp* ^3^ carbon domain in the ELA‐GO nanocomposite increased with the loading of GO [35].

### 5.9 Atomic force microscopy topographic imaging

**FIGURE 9:**
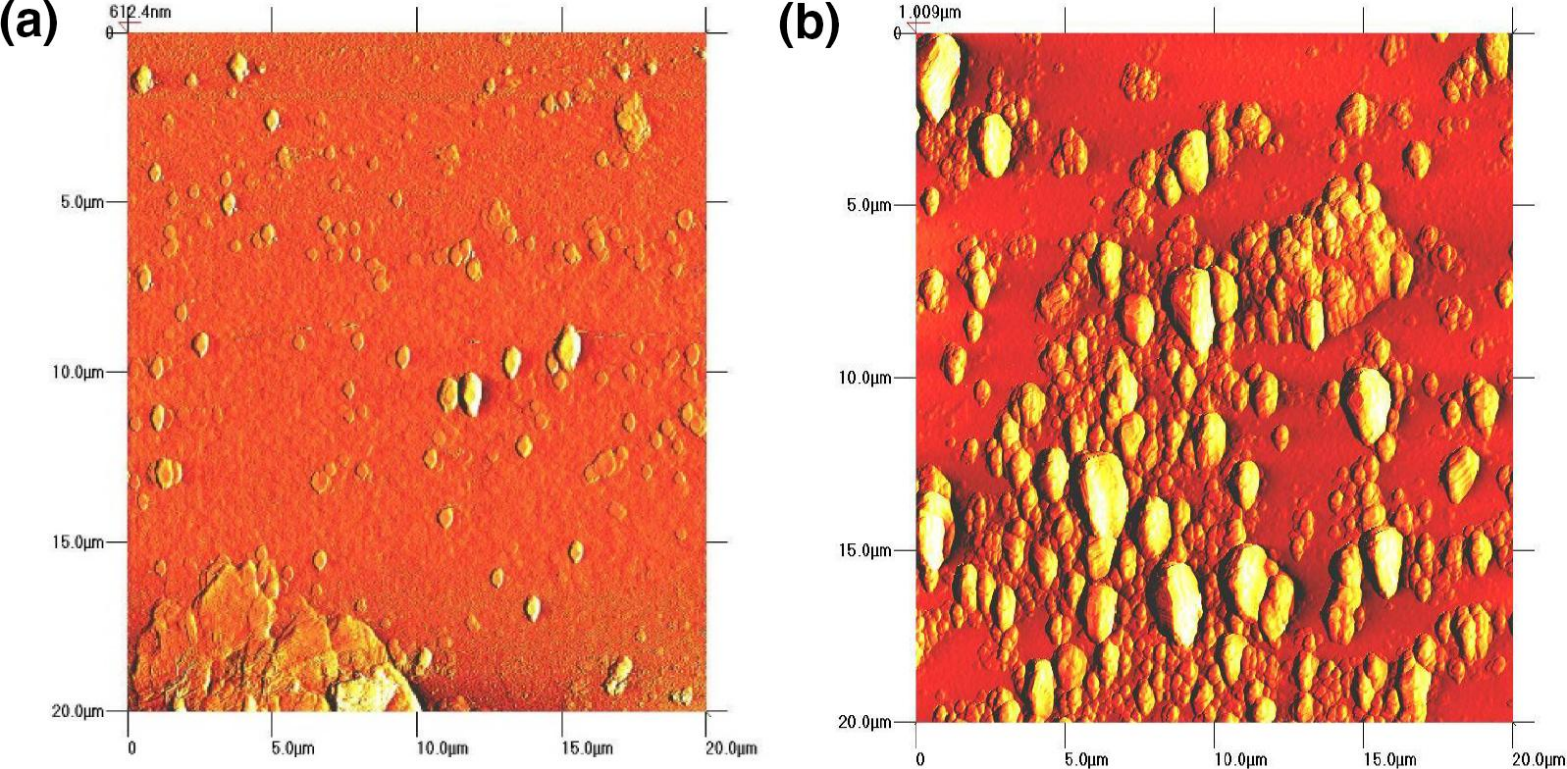
AFM images of GO (a) and ELA‐GO nanocomposite (b)

The vertical distance of GO and ELA‐GO is characterised by AFM. Figure 9a shows that the vertical distance of GO is about 1.3 nm [36], whereas the vertical distance of ELA‐GO nanocomposite (Figure 9b) is about 1.7 nm, which is larger than that of GO. This is due to the adsorption of ELA on both sides of GO [37].

### 5.10 In vitro release of ellagic acid from ellagic acid‐graphene oxide nanocomposite

**FIGURE 10:**
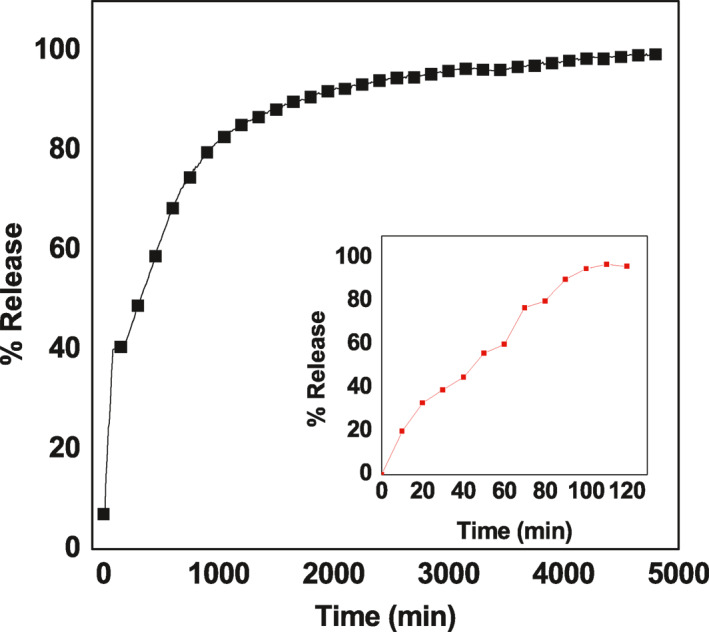
In vitro study of ellagic acid (ELA)‐gaphene oxide (GO) nanocomposite

To study the release actions of ELA from ELA‐GO nanocomposite and from physical form, the samples were dispersed in 0.01 M concentration of phosphate buffer solutions at pH 7.4. A signal was recorded at different time intervals, and the release curve for the physical form of ELA and nanocomposite is shown in Figure 10. This figure shows that ELA is quickly released from the physical form, and release is complete within 100 min in release media. The release rate of ELA from ELA‐GO nanocomposite was clearly slower than that from the physical form. This is accredited to the electrostatic attraction between ELA and the different functional groups of GO (–OH and –COOH groups).

Figure 10 show the release profiles for ELA for the 5000 min. It was found that the release is very quick for the first 50 min with 40% release at pH 7.4. This occurrence is most probably due to the ‘burst effect’ [38], which occurs due to the presence of free ELA in the sample which appeared in the PXRD pattern of the nanocomposite in Figure 1c. After the burst stage, a slower one followed, with a release value of 98% after 4850 min. This result indicates that ELA‐GO nanocomposite has potential as a controlled‐release formulation of drugs.

The successful formation of ELA‐GO nanocomposite was further confirmed by UV–Vis release study. The ELA loading percentage in the nanocomposite was analysed and showed that the value was 30%. The controlled release of ELA from the ELA‐GO nanocomposite was performed in 0.01 M concentration of sodium saline solution (Figure 10). The first burst release occurred at 50 min due to the presence of free ELA in the sample which appeared in Figure 1c. The ELA was released very slowly from GO with a 98% value after 4850 min [Figure 10]. This slow release was due to the hydrogen‐bonding interaction between –OH groups in ELA and the –COOH groups on GO [25,39].

The release rate of ELA from ELA‐GO nanocomposite is mostly due to the swelling‐diffusion mechanism. The d‐spacing can be described as the distance between planes of atoms that can be collected by an XRD instrument [40]. The d‐spacing of dry GO initially soaked in water was 0.76 nm. As the GO remained in water for a longer time, the d‐spacing increased and reached 6 to 7 nm at equilibrium. This result indicates a swelling of GO in aqueous solution [41]. From the literature, the cefadroxel was diffused from GO nanocomposites due to the exponent diffusion type value (*n*) which is between 0.5 and 1 [42]. According to this result, after the swelling process of GO, the diffusion mechanism of ELA at pH 7.4 occurs easily.

### 5.11 Drug release kinetics of ellagic acid

**FIGURE 11:**
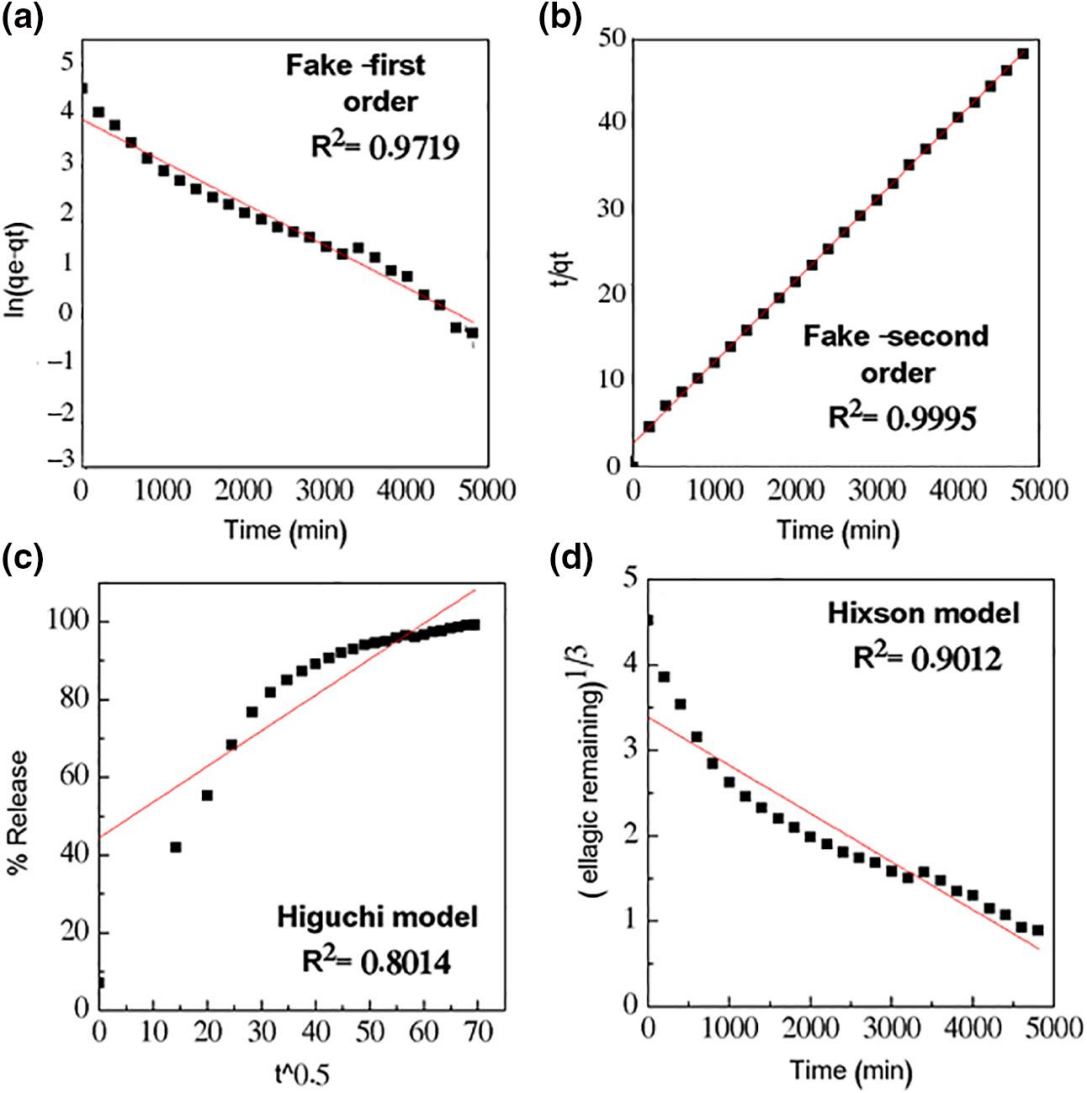
llagic acid (ELA) release from ELA‐graphene oxide (GO) nanocomposite data fitting

ELA release kinetics from the ELA‐GO nanocomposite is illustrated in Figure 11. In addition, the regression coefficient (*R* ^*2*^) values for ELA are tabulated in Table 1. The model that gave higher *R* ^*2*^ values was considered a best fit model. Based on the *R* ^*2*^ values, it was also observed that the release of ELA from ELA‐GO nanocomposite followed the fake‐second‐order kinetic model (Figure 11) [43].

**TABLE 1:** The *R* ^2^ values collected by fitting the ELA drug release data

Models	Equation	*R* ^2^
Fake‐first order	ln (q_e_ − q_t_) = ln q_e_ − k_1_t	0.9719
Fake‐second order	t/*q*_t_ = 1/k_2_q_e_^2^ + t/q_e_	0.9995
Higuchi	$q_{t}=K_{H}\sqrt{t}$	0.8014
Hixson‐Crowell	$\sqrt[{3}]{M_{o}}-\sqrt[{3}]{q_{t}}=Kt$	0.9012

Abbreviations: ELA, ellagic acid.

### 5.12 Cytotoxicity studies

**FIGURE 12:**
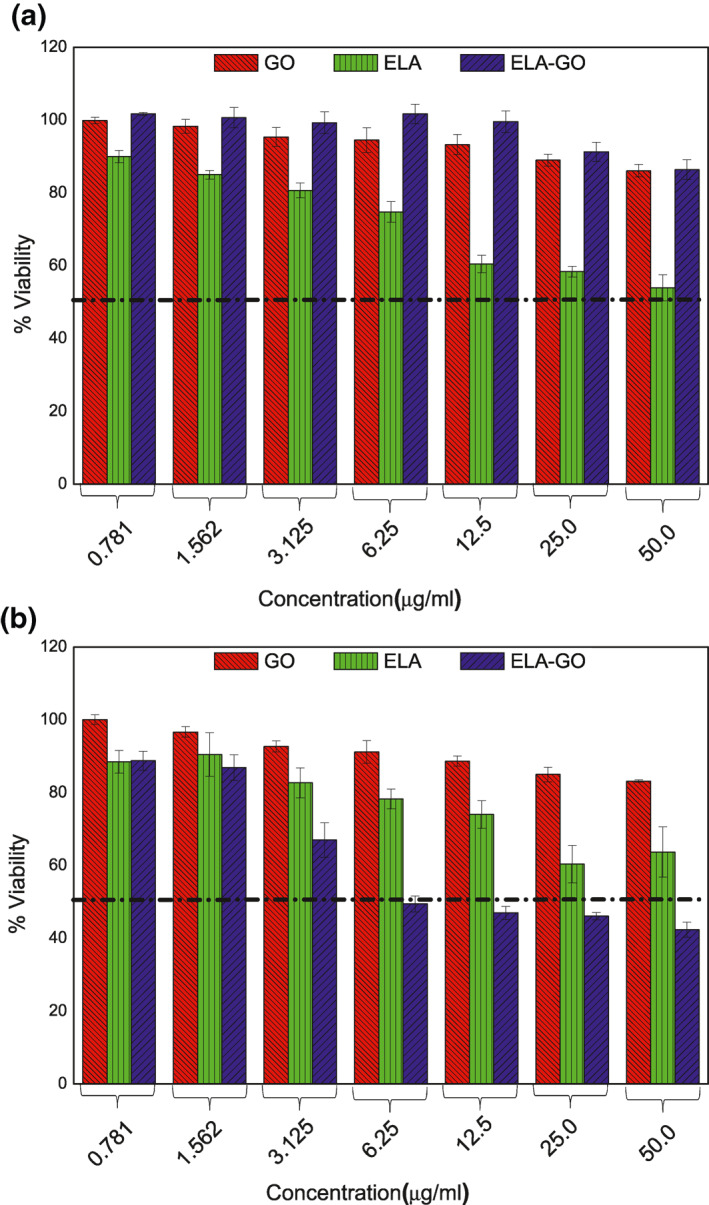
The cytotoxicity profiles for the ellagic acid (ELA), graphene (GO) and ELA‐GO nanocomposite after treatment of 3T3 cells (a) and B16 cells (b)

The cytotoxicity of ELA‐GO nanocomposite was compared to that of free ELA and GO on the B16 cancer cell line. In Figure 12b, only ELA‐GO nanocomposite produced a dose‐dependent cytotoxic effect on B16 cells after 24‐h treatment with an *IC*_50_ of 6.16 μg/ml. The cells’ viability decreased to about 42.4% at 50 μg/ml using ELA‐GO nanocomposite, compared to ELA and GO which showed 83.2% and 63.7% decrease in B16 cells’ viability, respectively. In the 3T3 cells (Figure 12a), no cytotoxicity effects at concentrations more than 50 μg/ml were observed, indicating that the ELA‐GO nanocomposite is safe to use in drug delivery.

### 5.13 Antimicrobial studies

The results in Table 2 show that the ELA‐GO nanocomposite at all tested concentrations (0.5, 1, and 5 mg/ml) have antibacterial activity towards both Gram positive and Gram negative bacteria in a concentration‐dependent manner except for 0.5 mg/ml which did not show activity against *P. aeruginosa*.

**TABLE 2:** Antimicrobial activity of ELA, GO and ELA‐GO nanocomposite

Microorganism	Diameter of zone of inhibition (mm)						
	5 mg/ml			1 mg/ml		0.5 mg/ml	
	EA‐GO	EA	GO	EA‐GO	EA	EA‐GO	EA
*Proteus mirabilis*	3.07 ± 0.089	1.65 ± 0.070	0	2.63 ± 0.044	1.1 ± 0.141	2.53 ± 0.057	0
*Escherichia coli*	2.77 ± 0.156	1.6 ± 0.141	0	2.43 ± 0.044	1.3 ± 0.141	2.23 ± 0.057	0
*Pseudomonas aeruginosa*	2.5 ± 0.067	0	0	1.56 ± 0.044	0	0	0
*Klebsiella pneumoniae*	3.3 ± 0.11	1.5 ± 0.141	0	2.93 ± 0.178	1.15 ± 0.070	2.83 ± 0.152	0
MRSA	2.87 ± 0.11	0	0	2.27 ± 0.156	0	2.07 ± 0.115	0
*Staphylococcus aureus*	2.97 ± 0.15	0	0	2.33 ± 0.111	0	2.17 ± 0.152	0

*Note*: The diameter of zone of inhibition for dimethyl sulfoxide (DMSO) was zero.

Abbreviations: ELA, ellagic acid; GO, graphene oxide; MRSA, methicillin‐resistant *S. aureus*.

The effect of ELA‐GO nanocomposite was more evident than ELA alone at the same tested concentration (5 mg/ml). ELA showed antibacterial activity against *P. mirabilis, E. coli,* and *K. pneumoniae*, and no activity was shown against Gram positive bacteria and *P. aeruginosa*. The GO (5 mg/ml) and DMSO did not show any antibacterial activity against all the tested bacteria. From these results, it can be deduced that the preparation of ELA‐GO nanocomposite will enhance the antibacterial activity of ELA against both Gram positive and Gram negative bacteria. Krishnamoorthy reported that the GO exhibited antibacterial activity against Gram positive and negative bacteria by mainly generating reactive oxygen species (ROS) which disturb the bacterial cells [44]. Ohemeng studied the effect of several flavones including ELA against the DNA gyrase activity of *E. coli*, and they found that the effect of ELA was evident [45]. Accordingly, this will highlight the importance of using nanocomposite of antimicrobial agents in order to reduce the required concentration of the active materials that are used as antimicrobials by obtaining high activity with less concentration.

## 6 CONCLUSION

In conclusion, this study confirmed the successful loading of ELA onto the surface(s) of GO. The nanocomposite was characterised by XRD, FTIR, TGA, TEM, SEM, AFM, and zeta‐potential. This nanocomposite could successfully affect B16 cells and lead to tumor cell inhibition with an *IC*_50_ of 6.16 μg/ml. In contrast, the ELA and GO alone did not show any inhibition to the same cells. The release of the ELA from ELA‐GO nanocomposite followed fake‐second order kinetic model. In addition, The of ELA‐GO nanocomposite was more evident than ELA alone at the same tested concentration (5 mg/ml).
